# Loss‐of‐function genetic screen unveils synergistic efficacy of PARG inhibition with combined 5‐fluorouracil and irinotecan treatment in colorectal cancer

**DOI:** 10.1002/ctm2.70543

**Published:** 2025-12-26

**Authors:** Cristina Queralt, Cristina Moreta‐Moraleda, Marta Costa, Ferran Grau‐Leal, Jeannine Diesch, Carla Vendrell‐Ayats, Eva Musulén, Roni H G Wright, Cristina Bugés, José Luis Manzano, Sara Cabrero‐de las Heras, Johannes Zuber, Marcus Buschbeck, Sonia‐V Forcales, Eva Martínez‐Balibrea

**Affiliations:** ^1^ CARE Program Germans Trias i Pujol Research Institute (IGTP) Badalona Spain; ^2^ ProCURE Program Institut Català d'Oncologia Badalona Spain; ^3^ Badalona Applied Research Group of Oncology (B‐ARGO) Medical Oncology Department Institut Català d'Oncologia Badalona Spain; ^4^ Cancer and Leukaemia Epigenetics and Biology Program Josep Carreras Leukaemia Research Institute (IJC) Campus ICO‐IGTP‐UAB Badalona Spain; ^5^ Hospital Universitari General de Catalunya‐Grupo Quironsalud Sant Cugat del Vallès Spain; ^6^ Cancer Epigenetics Group Josep Carreras Leukaemia Research Institute (IJC) Campus ICO‐IGTP‐UAB Badalona Spain; ^7^ Department of Biomedical Sciences Faculty of Medicine and Health Sciences Universitat Internacional de Catalunya (UIC) Barcelona Spain; ^8^ Research Institute of Molecular Pathology (IMP) Vienna BioCenter (VBC) Medical University of Vienna Vienna Austria; ^9^ Department of Pathology and Experimental Therapy Serra Húnter Programme Immunology Unit Faculty of Medicine and Health Sciences Universitat de Barcelona Barcelona Spain; ^10^ Immunity, Inflammation and Cancer Group Oncobell Program Institut d'Investigació Biomèdica de Bellvitge‐IDIBELL L'Hospitalet de Llobregat Spain; ^11^ Present address: Biomedical Sciences Department, International University of Catalonia Carrer Josep Trueta s/n (Hospital Universitari General de Catalunya) Sant Cugat del Vallès 08195 Spain.; ^12^ Present address: AIDS Research Institute‐IrsiCaixa Germans Trias i Pujol Research Institute (IGTP) Badalona 08916 Spain.

**Keywords:** 5FU, colorectal cancer, FUIRI, irinotecan, loss‐of‐function screening, PARG, PDD00017273

## Abstract

**Background:**

Colorectal cancer (CRC) remains a major global health concern, partly due to resistance to therapy and the lack of new effective treatments for advanced disease. The combination of 5‐Fluorouracil (5FU, a thymidylate synthase inhibitor) and irinotecan (a topoisomerase 1 inhibitor) is widely used in first‐line and subsequent treatments. This study aimed to identify novel therapeutic targets to enhance combinatorial therapy, improving treatment efficacy and durability of response.

**Methods:**

We performed a loss‐of‐function screen using HT29 CRC cell line and a retroviral library containing 7296 shRNAs targeting 912 chromatin genes. Cells were then treated with 5FU and SN38 (the active metabolite of irinotecan) or left untreated for 4 weeks. Genes enriched in resistant clones were identified through next‐generation sequencing. Amongst candidate genes, PARG was selected for functional validation.

**Results:**

CRISPR/Cas9‐mediated knockout (HT29 PARG‐KO) resulted in increased global poly(ADP‐ribosyl)ation after 5FU and SN38 treatment. PARG depletion led to reduced cell viability and increased apoptosis, particularly after 5FU exposure. Pharmacological PARG inhibition (PDD00017273) synergised with 5FU and SN38 across three CRC models (HT29, DLD1, HT115). In vivo, HT29 PARG‐KO xenografts were more sensitive to 5FU. Immunohistochemical analysis of 170 CRC patient tumours revealed that positive PARG expression correlated with poor response to 5FU + Irinotecan, increased liver metastases, and worse long‐term survival.

**Conclusions:**

Our findings highlight PARG as a promising therapeutic target for CRC, where its inhibition enhances the efficacy of standard chemotherapy.

**Key points:**

- LOF screening identified PARG as a modulator of CRC response to FUIRI treatment.
- HT29 PARG‐KO cells showed increased PAR, DNA damage, apoptosis, and 5FU sensitivity.
- Pharmacological PARG inhibition synergised with 5FU and SN38 in CRC cell lines.
- HT29 PARG‐KO tumours exhibited increased sensitivity to 5FU treatment in vivo.
- PARG expression in CRC tumours correlated with poorer responses and patient survival.

## Graphical abstract

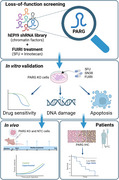

## 1 BACKGROUND

Colorectal cancer (CRC) is the most common tumour in both sexes worldwide, with serious social and economic repercussions. In recent years, the increasing incidence in individuals under 50 years of age has placed it under the spotlight. Overall survival (OS) at 5 years is around 65%, although in metastatic disease this does not exceed 15%. The treatment of choice in these cases has remained largely unchanged for more than two decades, consisting of combinations of classic chemotherapy (fluoropyrimidines, oxaliplatin, irinotecan) with an antiangiogenic agent or an EGFR inhibitor (only in patients whose tumours do not carry mutations in the RAS oncogenes). This approach has resulted in objective responses in approximately 50% of cases, with acquired resistance being the main cause of tumour progression in responders. Unfortunately, only about 5% of patients with metastatic CRC exhibit microsatellite instability‐high (MSI‐H) or mismatch repair deficiency (dMMR), which are the subgroups that currently benefit from immunotherapy. In light of this, it is imperative to identify new potential therapeutic targets that offer treatment alternatives, either in monotherapy or in combination with existing drugs.

The approval of the combination therapy comprising 5‐fluorouracil (5FU), leucovorin (LV), and irinotecan (FOLFIRI) for metastatic CRC in 2000 marked a significant advancement in treatment efficacy over the standard 5FU and LV regimen.^1^ Since its approval, FOLFIRI (and other schedules combining 5FU and irinotecan) has emerged as one of the cornerstones of the metastatic CRC therapy, alongside the alternative regimen of 5FU, LV, and oxaliplatin. 5FU represents an uracil analogue wherein a hydrogen atom in the carbon 5 position of the pyrimidine ring is substituted with a fluorine atom. Following a cascade of enzymatic transformations, 5FU undergoes conversion into active metabolites including fluorodeoxyuridine monophosphate (FdUMP), fluorodeoxyuridine triphosphate (FdUTP), or fluorouridine triphosphate (FUTP). These metabolites exert inhibitory effects on thymidylate synthase (TS) upon integration into DNA or RNA. Such inhibition disrupts the synthesis of dTMP, leading to perturbations in the deoxynucleotide pool, particularly altering the ratio of dATP to dTTP. Consequently, this dysregulation profoundly impairs DNA synthesis and repair mechanisms, culminating in cellular demise. Irinotecan, or CPT‐11, is a water‐soluble derivative of camptothecin, a natural plant alkaloid. Upon metabolism, irinotecan gives rise to SN38, which serves as a potent inhibitor of topoisomerase I (*TOP1*). SN38 specifically binds to the DNA‐topoisomerase 1 complex, obstructing DNA replication and inducing double‐stranded DNA breaks, thereby triggering apoptotic cell death. These actions predominantly occur during the DNA synthesis phase (S phase), rendering irinotecan a cycle‐specific cytotoxic agent.

Chromatin regulators are proteins and protein complexes that govern the structure and accessibility of chromatin. Their principal function lies in orchestrating gene expression modulation by finely tuning DNA accessibility to transcription factors and other regulatory proteins. This pivotal role extends to DNA repair processes, where they wield influence over the recruitment and activity of repair machinery at sites of DNA damage. Therefore, chromatin regulators can play a significant role in modulating the efficacy of 5FU plus irinotecan treatment in CRC with some of them being proposed as biomarkers of response and as possible therapeutic targets whose inhibition could result in synergistic treatments.^2^ Using a loss‐of‐function (LOF) genetic screen specifically designed to silence genes coding for chromatin regulatory factors, we aimed to identify potential therapeutic targets whose combination with 5FU + irinotecan results in synergistic treatment in CRC.

## 2 MATERIALS AND METHODS

### 2.1 Cell culture

HT29, DLD1 and HT115 human CRC cell lines were obtained from the American Type Culture Collection. Table S1A shows the list of mutations for each of the cell lines according to the data published in the Cellosaurus and COSMIC databases. Platinum‐E (PLAT‐E) and HEK293T cells were used for retro‐ and lentiviral production, respectively. HT29‐5FU resistant (R) cells were obtained as a result of continuous exposure to increasing concentrations of 5FU until a concentration of 2 µM was reached, as previously described.^3^ Cells were grown at 37°C and 5% CO_2_ in McCoy's 5A medium GlutaMAX (Lab Clinics) (HT29/HT29‐5FUR), RPMI 1640 medium (LabClinics) (DLD1 and HT115) and DMEM/F12 medium (Invitrogen) (Platinum‐E (PLAT‐E) and HEK293T) supplemented with 10% fetal bovine serum (FBS) (Reactiva) and 1% Penicillin–Streptomycin–Amphotericin B (Invitrogen). Additionally, RPMI and DMEM/F12 media were supplemented with 10 mM glutamine (Invitrogen), and 10 mM HEPES (Thermo). Cells were periodically tested for Mycoplasma contamination and authenticated by short tandem repeat profiling using in‐house methods. All experiments were conducted using cells with fewer than 10 passages to ensure reproducibility.

### 2.2 Drugs

5FU and irinotecan were obtained from leftover vials prepared for patients at the pharmacy of the Catalan Institute of Oncology. SN38, the active metabolite of irinotecan, and the PARG inhibitor PDD00017273 (hereafter referred to as PDD) were purchased from MERCK and TOCRIS Bio‐Techne, respectively. The purity of PDD00017273 is more than 98% analysed by HPLC (https://www.tocris.com/products/pdd‐00017273). COH34 PARG inhibitor was provided by MedChemExpress with a purity of 99.92% (https://www.medchemexpress.com/). PDD and COH34 were reconstituted in Dimethyl sulfoxide (DMSO) to obtain a stock concentration of 5 and 10 mM, respectively, and stored at −20 °C. Further dilutions of each drug were made in the culture medium.

### 2.3 Lentiviral production

This procedure was employed to introduce the ECO receptor (pWPXLd‐rtTA3‐IRES‐EcoRec‐PGK‐Puro construct) for biosafety reasons and to generate a knockout (KO) model for PARG using the CRISPR/Cas9 technique, both in the HT29 cell line. The viral particles were generated by adding to HEK293T cells the packaging plasmids psPax2 and pCMV‐VSV‐G (both from Addgene) following Moore vector ratio (1:1:1) using Lipofectamine 2000 (Invitrogen) according to manufacturer's protocol. After 48 h of transfection, viral supernatant was filtered in 0.45 µm filters (Merck‐Millipore) and added to HT29 cells at 70% of confluence in the presence of 8 µg/µL of Polybrene (Merck‐Sigma Aldrich). 72 h later, HT29 cells were selected for at least 1 week with 0.35 µg/mL puromycin (Merck‐Sigma Aldrich). The introduction of the Eco receptor into our cells was confirmed through qPCR (Figure S1). The primers used are listed in Table S2.

### 2.4 CRISPR/Cas9 PARG‐KO

HT29 cell line was infected with lentiviral particles containing a pool of three different gRNA for the PARG gene (pLentiCRISPR v2; GenScript) generated independently following the previously described protocol (Table S3). After 48 h‐production of individual gRNA lentivirus, independently viral supernatants were filtered and mixed in a 1:1:1 proportion doing a pool. 1/40 dilution of the viral mix was added to HT29 cells at 70% of confluence in a proportion of 1:3 of total plate medium volume. At 4‐ and 24‐h post‐infection 1:3 of fresh media was added. After 1 week of puromycin selection, single‐cell sorting was performed by FACSAria II (BD Biosciences) to isolate homogenous edited cells within the mixed pooled edited population. The clonal edition was confirmed by Sanger sequencing (see primers in Table S2). A negative non‐target control (NTC) (NonTargetingControl Guide for Human, GenScript) was also infected in the HT29 cell line following the same procedure. The resulting cell lines were named HT29 PARG‐KO and HT29 NTC, respectively.

### 2.5 Retroviral production

This method was used to infect HT29 EcoR cells with the shRNA hEPI9 library and to introduce to HT29 cells the individual shRNA for validation. The retroviral particles were generated by adding to Plat‐E cells the shRNA hEPI9 library or individual shRNA (both cloned in pMSCV‐LENC vector following standard cloning techniques) using Lipofectamine 2000 (Invitrogen), following manufacturer's protocol. After 72 h, viral supernatant was collected and filtered using 0.45 µm filters (Merck‐Millipore). A proportion of 1:3 of total plate medium volume of viral supernatant was added to cells at 70% of confluence in the presence of 8 µg/µL of Polybrene (Merck‐Sigma Aldrich). After 4 h post‐infection, up to 2:3 of fresh medium was added. 72 h post‐infection, cells were selected with 600 µg/mL of Geneticin (Thermo) for at least 1 week. The percentage of cell infection was measured by Flow Cytometry using mCherry cells positivity in a LSRFortessa SORP Flow Cytometer (BD Biosciences). For the subsequent individual validation of PARG, selected shRNAs and a non‐human shRNA control (shRenilla coming from firefly) was also infected following the same procedure.

### 2.6 Retroviral shRNA hEPI9 library infection and loss‐of‐function screening

The miR‐E‐based short hairpin library, hEPI9, comprises 7296 shRNAs targeting 912 chromatin genes (with 8 shRNAs per target) including both positive and negative shRNA controls. The functions of hEPI9 targeted genes were represented in Figure S2. This library was kindly provided by Dr. Johannes Zuber.^4^, ^5^ A retroviral infection efficiency of 1% was observed in previous experiments using HT29 EcoR cells. Since the hEPI9 library contains close to 7,300 shRNAs and we wanted to achieve a 1,000X representation of each one, 730 × 10^6^ HT29 EcoR cells were infected with the library retroviral supernatant following the protocol described above. After selection, cells were treated or not using the IC_20_ of FUIRI (0.1 µM of 5FU + 0.055 nM of SN38), 24 h after seeding. The next day, the medium was changed, and cells were allowed to grow for 72 h. This schedule was repeated four consecutive times for 3 weeks. After 21 days of treatment, cells were collected and prepared for sequencing.

### 2.7 Drug–response analysis by the 3‐(4,5‐dimethylthiazol‐2‐yl)‐2,5‐diphenyltetrazolium bromide (MTT) assay

To calculate the inhibitory concentrations (IC) of different drugs and combinations, we used the MTT assay (Roche) as previously described (3). Cells were seeded in 96‐well microtiter plates (Nunc) at densities of 1,500 cells per well for HT29 and DLD1, and 5,000 cells per well of HT115 cell line, incubated for 24 h and treated with a range of serial drug concentrations for 24 h. Subsequently, the drug‐containing media were removed and replaced with fresh media, allowing the cells to grow for 72 h. MTT was then added to each well and, following incubation, the resulting formazan crystals were solubilised using a mixture of 0.02 M HCl and 20% sodiium dodecyl suplphate (SDS) (50% v/v). The absorbance was measured at 570 nm using the SPECTROstar Nano spectrophotometer (BMG Labtech). The drug concentrations corresponding to each fraction of survival (ranging from 10% to 90% cell viability) were determined using the median‐effect line method. The maximum concentration of DMSO (vehicle) used was 0.5%, which did not affect cell proliferation in our experiments.

For the LOF screen, we established the FUIRI IC_20_: HT29 EcoR cells were treated with serial dilutions ranging from 1/6 to 1/100 of 5FU and SN38 IC_50_ in 1:1 proportion for 24 h‐treatment following the MTT assay. FUIRI IC_20_ (0.1 µM of 5FU + 0.055 nM of SN38) was determined after four consecutive 24‐h treatments (Figure S3) mimicking the schedule followed for LOF screen.

For the proliferation assay comparing the effect of PDD addition in HT29 NTC and PARG‐KO cells, cells were treated with 5 µM PDD 24 h after seeding. At each selected time point, MTT reagent was added and absorbance was measured.

### 2.8 Analysis of combined drug effects

Drug interaction was evaluated using the Chou–Talalay method, which is based on the median‐effect principle derived from the mass‐action law. In this approach, one drug can be tested at a fixed dose whilst the other is diluted in proportional ratios; alternatively, both drugs can be combined at constant ratios, as in our case, where each drug was diluted in parallel according to the same fractions of their respective IC50 values. This allows for the quantitative assessment of drug interactions. The combination index (CI) was then calculated, where CI < 1 indicates synergism, CI = 1 denotes an additive effect, and CI > 1 suggests antagonism. Dose–response curves for each drug alone and in fixed‐ratio combinations were generated, and CI values were determined using CompuSyn software. Cells were seeded at the corresponding cell density in a 96‐well plate, incubated for 24 h and treated with a range of serial dilutions of the previously established IC_50_ values for 5FU and SN38 for 72 h. FUIRI combinations (5FU + SN38) were prepared by mixing 5FU and SN38 at a 1:1 ratio of their IC_50_ values, and serial dilutions were made from 1/16 to 4 times the IC_50_. Additionally, PDD was added at final concentrations of either 1 µM or 5 µM.

### 2.9 Next generation sequencing Solexa technology

DNA was extracted using the phenol–chloroform–isoamylalcohol protocol and sent for sequencing by NGS (Illumina HiSeq2500 platform) to Dr. Zuber's lab. The sequencing results were analysed using the R package EdgeR (R Core Team 2019) as described,^6^ which calculated log‐fold change (logFC) values, counts per million, significance values (*p*‐values), and false discovery rates (FDR). Genes were selected if at least 6 out of 8 hairpins exhibited consistent behaviour in the same direction and were ranked on a gene‐by‐gene basis according to mean logFC, *p*‐value, and FDR values using the Roast function.

### 2.10 Gene expression by real time‐quantitative PCR

RNA was extracted from cultured cells using the Maxwell 16 LEV simplyRNA Cells Kit (Promega) according to the manufacturer's protocol in Maxwell RSC (Promega) supply. cDNA synthesis was performed with 1 µg of RNA using the SuperScript IV First‐Strand Synthesis System (Thermo) following the manufacturer's instructions. Quantitative PCR (qPCR) was carried out using pre‐designed IDT assays and SYBR Green master mix (Roche) in a LightCycler 480 II instrument (Roche) (Table S2). To ensure the absence of genomic DNA contamination, RT minus controls (no reverse transcriptase) were included for each sample, with contamination acceptable if below 5% of the total signal. All qPCR reactions were conducted in triplicates to ensure reproducibility and reliability of the results. Relative gene expression levels were calculated using the 2^(−ΔΔCt) method, with gene expression normalised to the reference gene β‐actin (IDT) and cells transfected with the control vector used as reference samples to determine relative expression levels. Data were analysed using LightCycler 480 software version 1.5 (Roche) for threshold cycle (Ct) determination.

### 2.11 Cell viability assay

Cells were seeded in 6‐well plates to confirm the effect of gene knockdown (KD) or KO upon treatment. Cells were treated for 24 h with serial dilutions (1/100, 1/50, 1/6, and 2X) of the combination of FUIRI IC_50_ as previously established by the MTT assay. After 24 h of treatment, the drug‐containing medium was removed, and the cells were allowed to grow for an additional 72 h. Subsequently, cells were collected along with the corresponding supernatant and centrifuged. Pellets were resuspended in 1X PBS and stained for 30 min at 37°C with 10 µM DiOC (Thermo). Following two washes with 1X PBS, cells were stained for 15 min with 3 µM DAPI (Merck‐Sigma Aldrich) in 1X PBS. Fluorescence levels were measured by flow cytometry using the LSR Fortessa SORP Flow Cytometer (BD Biosciences) and analysed with BD FACSDivaTM software (BD Biosciences).

### 2.12 Sanger DNA sequencing

DNA was extracted using the Maxwell RSC Cultured Cells DNA Kit (Promega) according to the manufacturer's protocol. 50 ng of DNA were then amplified by PCR with Phusion High‐Fidelity DNA Polymerase (2 U/µL) (Thermo) and specific primers (IDT) for 30 cycles (Table S2). Following amplification, the PCR product was verified for correct band size by 1% agarose gel electrophoresis. The product was purified using ExoSAP‐IT PCR Product Cleanup Reagent (Thermo) as per the manufacturer's instructions. Finally, the samples were sent for Sanger sequencing at Macrogen Inc.

### 2.13 Apoptosis assays

HT29 NTC and PARG‐KO cells were treated 24 h after seeding in 6‐well plates with serial dilutions (1/18, 1/3, 1X, and 2X) of the combination of individual IC_50_ values for 5FU and SN38 or the FUIRI combination. After 72 h of treatment, cells were collected along with the supernatant and centrifuged. Following two washes with cold 1X PBS, the cell pellets were resuspended in 1X Binding Buffer (BD Pharmingen) and stained for 15 min at room temperature, protected from light, with 4 µL of FITC Annexin V and 3 µL of Propidium Iodide (BD Pharmingen). Prior to measuring fluorescence levels using flow cytometry with the LSR Fortessa SORP Flow Cytometer (BD Biosciences), 400 µL of 1X Binding Buffer (BD Pharmingen) was added to the cells. The results were analysed with BD FACSDiva software (BD Bioscience).

### 2.14 Total and acidic histone‐enriched protein extraction

Total protein extraction began with the homogenisation of dry frozen cell pellets in RIPA buffer (PBS; NP‐40 1%; Na deoxycholate 0.5%; SDS 0.1%; EDTA 1 mM; NaF 50 mM; NaVO_3_ 5 mM) supplemented with a cocktail of EDTA‐free protease inhibitors (Roche) using the gentleMACS Dissociator system (Miltenyi Biotec). After a 15‐min incubation at 4°C, the samples were centrifuged at maximum speed for 15 min at 4°C. The aqueous supernatant containing the total protein extract was quantified using the Pierce BCA Protein Assay Kit (Thermo) according to the manufacturer's protocol. For histone isolation, the remaining pellets were resuspended in 0.2 M HCl, homogenised with the gentleMACS Dissociator system (Miltenyi Biotec), rotated at 4°C for 15 min, and then neutralised by adding Tris‐HCl (1 M; pH 8).

### 2.15 Western blot

Fifty micrograms of total protein or 25 µL of histone proteins were loaded onto NuPAGE 10% Bis‐Tris Midi Protein Gels (Invitrogen) and run using 2‐(N‐morpholino)ethanesulfonic acid SDS running buffer 1X (Invitrogen), at 180 V for 1 h. Gels were dry transferred to polyvinylidene fluoride membranes using the iBlot 2 Dry Blotting System (Thermo). After transferring, membranes were blocked for at least 1 h at room temperature with Intercept (TBS) Blocking Buffer (LI‐COR, Invitrogen). Membranes were then incubated with specific primary antibodies overnight at 4°C. The following day, after several washes with 1X TBS‐Tween 0.1%, membranes were incubated with secondary LI‐COR antibodies for 1 h at room temperature, protected from light. After several washes with 1X TBS‐Tween 0.1%, membranes were scanned and analysed using the LI‐COR Odyssey 9120 Digital Imaging System. Band quantification was performed using Image Studio Software and normalised by comparing phosphorylated proteins to their non‐phosphorylated counterparts or to a reference protein such as α‐tubulin. The antibodies used for WB were: 1/1,000 PARG mouse antibody (OTI6F4) (NBP2‐46320; Novus); 1/20,000 α‐tubulin mouse antibody (T6074; Sigma); 1/1,000 Anti‐Histone H2A.X rabbit antibody (AB11175; Abcam); 1/1,000 Anti‐phospho‐Histone H2A.X (Ser139) mouse antibody clone JBW301 (05‐636; Merck); and 1/1,000 PAR mouse antibody (AM80; Sigma), and 1:500 anti‐cleaved Caspase3 antibody (AB32042; Abcam).

### 2.16 Immunofluorescence

HT29 NTC and HT29 PARG‐KO cells were seeded in LabClinics chambers. After 24 h, cells were treated with a 3.33 µM 5FU or 3.33 µM 5FU + 10 nM SN38 adding PDD at 5 µM alone or in combination with 5FU and FUIRI for 24 h. Following treatment, the media were replaced with formaldehyde (4% in TBS) for fixation. Permeabilisation was performed using 0.2% Triton X‐in 1X TBS, and blocking was done with 1% BSA in 1X TBS for 1 h. Cells were then incubated with a 1:100 dilution of γH2A.X antibody (05‐636; Merck) or 1:150 dilution of PAR anibody (4335‐MC‐100; R&D Systems) in freshly prepared 1% BSA‐1X TBS overnight. After several washes, cells were stained with a 1:1,000 dilution of fluorophore‐conjugated Goat anti‐Mouse Alexa 555 secondary antibody (A‐21422; Invitrogen) or fluorophore‐conjugated Goat anti‐Mouse Alexa 488 secondary antibody (A‐11001; ThermoFisher Scientific) in freshly prepared 1% BSA‐TBS for 1 h, protected from light. Following additional washes, cells were stained with a 1:20,000 dilution of 0.5 mg/mL DAPI (Merck‐Sigma Aldrich) for 5 min to visualise the nucleolus. Slides were mounted with ProLong Gold Antifade (Thermo). Images were captured using the Micro Axio Imager M2 Apotome (Zeiss) and the total γH2AX foci/field were quantitated by the Find Maxima feature of ImageJ software. At least 200 nuclei were analysed per sample.

### 2.17 Measurement of protein‐bound DNA using the advanced recovery of K⁺‐SDS precipitates (ARK) method

HT29 NTC and PARG‐KO cells were lysed following the protocol described by Hu et al.,^7^using a buffer containing the chaotropic agent guanidine thiocyanate (GTC) and detergents to ensure stringent conditions that disrupt non‐covalent DNA–protein interactions, which represent a major source of background. Cell lysis (5.6 M GTC; 10 mM Tris‐HCl, pH 6.5; 20 mM EDTA; 4% Triton X‐100; 1% sodium N‐lauroylsarcosinate; 1% DTT) was performed at 55°C to further increase stringency and minimise nonspecific binding. The resulting genomic DNA, including DNA–protein crosslinks (DPCs), was precipitated with 50% ethanol and washed (20 mM Tris‐HCl, pH 6.5; 150 mM NaCl; 50% ethanol). The pellet was resuspended (1% SDS; 20 mM Tris‐HCl, pH 7.5) at a denaturing temperature to dissociate residual non‐covalent complexes. Subsequently, a KCl‐mediated precipitation step (200 mM KCl; 20 mM Tris‐HCl, pH 7.5) was applied to remove free DNA, and the protein‐associated DNA was released by proteinase K digestion (100 mM KCl; 20 mM Tris‐HCl, pH 7.5; 10 mM EDTA; 0.2 mg/mL proteinase K). DNA concentration was determined using the PicoGreen assay, and DPC levels were calculated as the ratio between protein‐bound DNA and total genomic DNA.

### 2.18 In vivo experiments

Experiments were designed as exploratory range‐finding studies using minimal group sizes, in accordance with international guidelines and the 3Rs principle, to ensure animal welfare whilst confirming safety at sub‐therapeutic doses. Although OECD guidelines were originally developed for oral administration, their principles have been broadly applied to other routes, including intraperitoneal (IP) injection. Based on our previous experience in preclinical mouse models, the IP route was chosen over intravenous (IV) administration as it ensures accurate dosing and reduces animal stress associated with immobilisation. Whilst several studies have compared sequential versus concomitant administration of 5FU and CPT‐11, the simultaneous regimen was selected following Guichard and colleagues,^8^ who demonstrated that concomitant dosing achieves an optimal balance between efficacy and tolerability. The doses used in this study (50 mg/kg 5FU and 50 mg/kg CPT‐11) were selected according to published preclinical studies reporting maximum tolerated doses higher than those used here and demonstrating efficacy in CRC xenografts.^8^, ^9^, ^10^, ^11^ These doses therefore represent a conservative yet therapeutically relevant regimen consistent with previous in vivo work.

Five‐week‐old athymic nude Foxn1^nu^ male and female mice were purchased from Envigo and acclimated in a temperature‐ and humidity‐controlled room at the Center for Comparative Medicine and Bioimage (CMCiB) on a 12‐h light–dark cycle for 1 week prior to the experiment. Mice were housed under specific pathogen‐free conditions and monitored according to strict welfare protocols adhering to the 3Rs principle. A preliminary tumour growth and toxicity test was conducted before to evaluate treatment efficacy. Tumour growth was assessed by subcutaneously injecting 3 × 10⁶ of HT29 NTC or PARG‐KO cells into the left flank of each mouse (100 µL) in McCoy's 5A medium GlutaMAX (LabClinics) mixed with Matrigel Matrix Basement Membrane (Corning) at a 1:1 ratio into 10 mice per group (5 females and 5 males each). Tumour growth was measured twice a week using a digital caliper. Tumour volume (TV) was calculated using the formula *V* = 1/2 × length (mm) × [width (mm)]^2^ over 21 days. Treatment toxicity was assessed as follows: 10 mice were treated IP once a week for 4 weeks with either PBS 1X (*n* = 2), 50 mg/kg CPT‐11 (*n* = 2), 50 mg/kg 5FU (*n* = 5), or a combination of 50 mg/kg 5FU + 50 mg/kg CPT‐11 (FUIRI) (*n* = 10). Toxicity was assessed by weekly monitoring of body weight. A suspension of 2.5 × 10^6^ HT29 NTC or HT29 PARG‐KO cells in McCoy's 5A medium GlutaMAX (LabClinics) mixed with Matrigel Matrix Basement Membrane (Corning) at a 1:1 ratio was injected subcutaneously into the left flank of each mouse (100 µL). Tumour growth was measured twice a week using a digital caliper, and TV was calculated with the formula previously described. Once tumours reached 100 mm^3^, mice were treated IP with vehicle (PBS 1X), 50 mg/kg 5FU, 50 mg/kg CPT‐11, or FUIRI (50 mg/kg 5FU + 50 mg/kg CPT‐11) once per week for 4 weeks, according to their treatment group. Relative tumour volume (RTV) was determined by the ratio of each measurement's TV to the TV on the first day of treatment for each group. Tumour growth inhibition (TGI) was calculated using the formula TGI (%) = [1 − (RTV of the treated group)/(RTV of the control group)] × 100 (%). At the end of the experiment, mice were fully anaesthetised and euthanised by cervical dislocation. To determine the appropriate number of animals per group, a power analysis was performed using the online calculator available at https://www.stat.ubc.ca/~rollin/stats/ssize/n2. Assuming a moderate effect size (Cohen's *d* ≈ 1) and a power of 0.8, the minimum number of animals required was estimated at seven per group. To ensure sufficient statistical robustness and account for potential biological variability, 10 mice per group were used in all experiments. The Animal Experimentation Commission of the Catalan Government approved all in vivo procedures under project reference 11730.

### 2.19 PARG immunohistochemical analysis

We used six tissue microarrays (TMAs) constructed from the FFPE primary tumours of 170 metastatic CRC patients who received either irinotecan (180 mg/m^2^ on day 1) + 5FU (400 mg/m^2^ bolus and 600 mg/m^2^ 22‐h infusion) + leucovorin (LV) (200 mg/m^2^ on days 1–2) (FOLFIRI) every 2 weeks or irinotecan (80 mg/m^2^) + 5FU (250 mg/m^2^ 48‐h infusion) (FUIRI) weekly, as first‐line treatment, in the framework of a clinical study.^12^ The Hospital Germans Trias i Pujol Ethics Committte approved the study with reference number PI‐19‐105. All patients signed an informed consent to participate in the study. Patient characteristics are in Table 1.

**TABLE 1:** PARG inhibitors.

Name	IC50	Characteristics	Ref.	Model
Gallotannin	16.8 µM	Low activity off‐target effects	Tsai et al., 1991	Preclinic
Rhodamine‐based PARG inhibitors	1–6 µm	Selective not cell‐permeable	Finch et al., 2012	Preclinic
GPI	1.7 µM	Low activity off‐target effects	Lu et al., 2003	Preclinic
ADP‐HPD	120 nM	Selective lack of cell permeability	Slama et al., 1995	Preclinic
PDD00017273	26 nM	Selective cell permeable low bioavailability	James et al., 2016	Preclinic
COH34	0.37 nM	Selective high efficiency of inhibition cell permeable	Chen and Yu, 2019	Preclinic
JA2131	400 nM	Selective cell permeable could be modified to increase inhibition potency	Houl et al., 2019	Preclinic
IDE161	N/A	Selective high efficiency of inhibition high preclinical tolerability profile	Monah et al., 2023	Phase 1 NCT05787587
ETX‐19477	Low nM	Selective high efficiency of inhibition well tolerated robust dose‐dependent in vivo anti‐tumour activity	Holleran et al., 2024	Phase 1 NCT06395519

*Note*: Most commonly used PARG inhibitors. All of these inhibitors have been evaluated in vitro to assess their efficacy. Notably, the last two inhibitors are currently being tested in Phase I clinical trials for solid tumours including CRC. N/A: not applicable.

Five micrometres thick TMA sections were analysed using standard IHC techniques. PARG expression was detected using a PARG mouse antibody (OTI6F4) (NBP2‐46320; Novus). The process was performed automatically using a Ventana BenchMark ULTRA machine (Roche). An external positive control was included on each slide. Immunostaining was independently evaluated by two pathologists. PARG expression was assessed using a four‐level scoring system: null (0), mild (1+), medium (2+), and strong (3+).

### 2.20 Statistical analysis

Statistical analyses of the experimental data were performed using the GraphPad Prism software (version 8). The specific statistical test used for each experiment is specified in the corresponding figure legend. Data visualisation includes results from at least three independent experiments, with figures showing mean values along with either standard deviations (SD) or standard error of the mean (SEM) as indicated. To evaluate significant differences between groups, Student's *t*‐test analysis and two‐way analysis of variance (ANOVA) were used, when appropriate. Statistical significance was defined as *p* < 0.05. For in vivo experiments linear mixed effects (LME) model was used to analyse the effect of treatments. Data from the patients study were analysed using PASW Statistics (version 18 for Mac), with the tests used specified in each figure caption. OS data are represented as Kaplan–Meier curves and the *p*‐value was calculated using the log‐rank test.

## 3 RESULTS

### 3.1 Genetic loss‐of‐function screening identifies PARG as a potential combinatorial drug target in colorectal cancer

**FIGURE 1:**
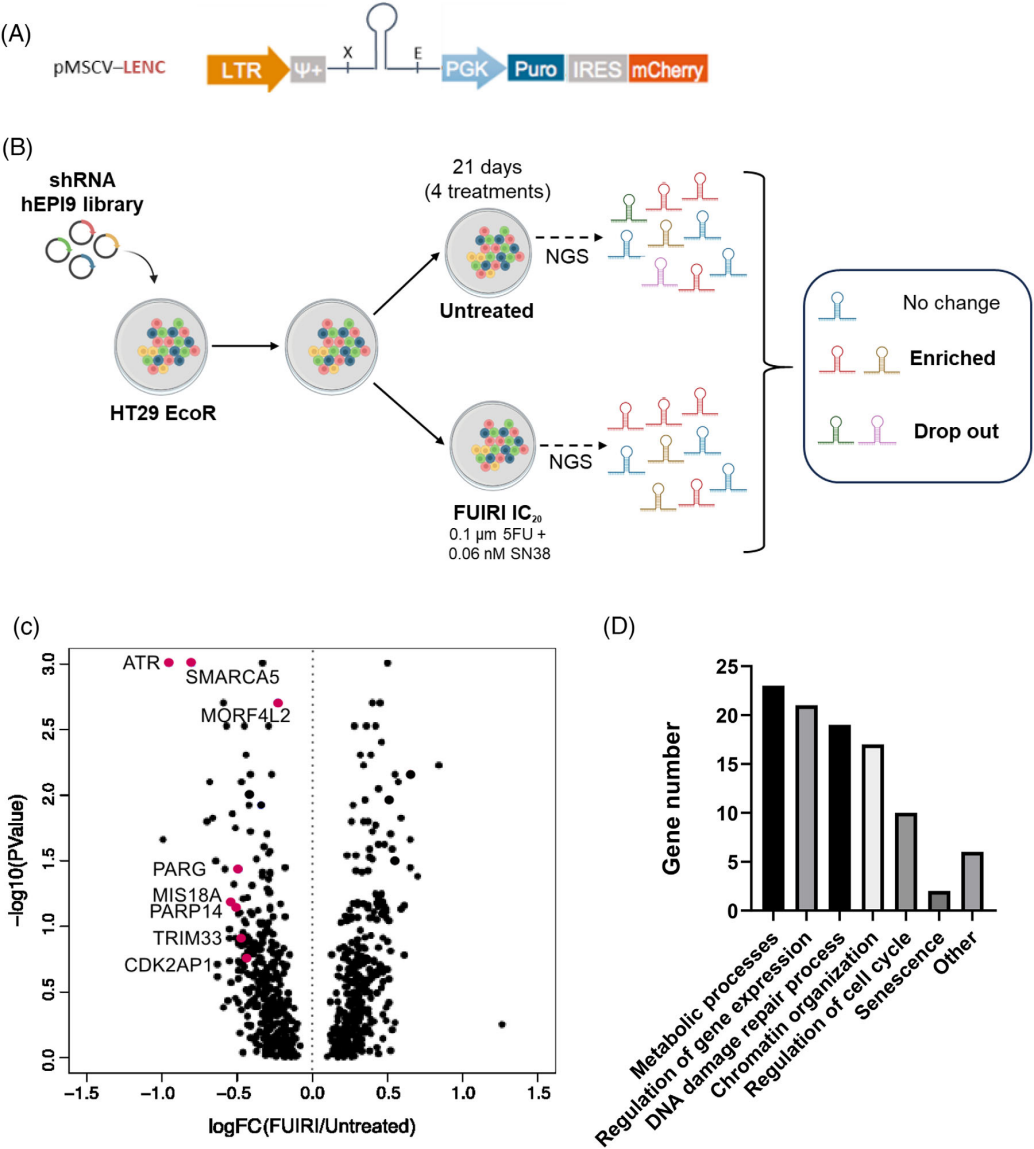
LOF screening design and results. (A) pMSCV vector. Diagram illustrating the elements of the pMSCV vector including a long terminal repeat (LTR) for integration, an extended retroviral packaging signal (Ψ), a shRNA integration site, a constitutive phosphoglycerate kinase promoter (PGK), a puromycin resistance gene (Puro) and an internal ribosomal entry site (IRES) for transcription and red fluorescent protein (mCherry). (B) LOF screening timeline. The HT29 EcoR cell line was infected with the hEPI9 library and treated with the IC_20_ of FUIRI for four consecutive treatments over 21 days. NGS was used to compare the shRNA representation between treated and untreated cells. (C) Volcano plot of candidate hits from the LOF screening. Of the 912 chromatin factors tested, 352 genes were significantly depleted, potentially indicating an association with FIUIRI sensitivity (left side of the volcano plot). In contrast, 131 genes were enriched suggesting a possible association with a lack of response to FUIRI treatment (right side of the volcano plot) based on logFC. Genes associated with FUIRI sensitivity, validated individually, are represented by red dots. (D) Gene Ontology (GO) analysis of the biological processes related to the depleted genes. GO analysis was performed using the PANTHER overrepresentation test, applying Fisher's exact test without any statistical correction. LOF, loss‐of‐function.

We used the shRNA hEPI9 library to perform a genetic LOF screening in a pooled approach to identify chromatin‐related genes that synergise with FUIRI treatment. An optimised mirE backbone was used to clone all shRNAs, increasing KD efficiency of target genes after single‐copy integration^4^, ^5^ (Figure 1A). As a cellular model, we chose the human CRC cell line HT29, which is well‐characterised in our laboratory.^13^, ^14^, ^15^ According to the COSMIC and Cellosaurus databases, these cells harbour mutations in key CRC‐associated genes *APC* (p.E853*), *BRAF* (p.Val600Glu), *PIK3CA* (p.Pro449Thr), and *TP53* (p.Arg273His), and are classified as CMS3^16^, ^17^ (Table S1A,B). The selected shRNAs target human chromatin‐related genes, so the cells were modified by introducing a murine ecotropic receptor via lentiviral infection (Figure S1). This strategy prevents harmful effects for the researcher, as these cells can only be infected by murine retrovirus that recognises this receptor. For the LOF screening, HT29‐EcoR (hereafter HT29) cells were infected with the hEPI9 shRNA library at low viral titter to favour single‐copy integration. After geneticin selection, cells were treated four consecutive times over 3 weeks with a combination of 5FU and SN38 at their corresponding IC_20_ (0.1 µM +.055 nM, respectively) (Figure S3). In mild treatments like IC_20_, shRNAs that drop out can pinpoint the most vulnerable genes. After this period, the pool of shRNAs integrated into the genomic DNA was compared between treated and untreated cells via NGS (Figure 1B). Potential candidate genes were selected based on the combination of different parameters such as the trend consistency of the shRNA (genes that showed the down representation of at least five out of eight shRNAs compared to untreated cells), the mean of the log fold change of the eight shRNA (the more negative values indicated higher sensitivity to treatment), the mixed *p*‐value of the eight shRNA (*p* < 0.1) and the FDR (< 0.5).

Using this approach, we identified 352 genes whose shRNAs were significantly depleted and 131 genes whose shRNAs were enriched, represented in the volcano plot in Figure 1C. The 24 top drop‐out genes identified are listed in Table S4. A decrease in shRNAs in treated cells indicated that KD of the corresponding genes was not tolerated, suggesting that the gene products are essential for survival in the presence of the FUIRI treatment. A Gene Ontology (GO) analysis was performed to identify the most common functions of the potential candidate genes (Figure 1D). Genes associated with sensitivity to FUIRI treatment were primarily involved in the regulation of metabolic processes and gene expression, regulation of DNA damage response (DDR) and chromatin remodelling as well. Based on a combination of published evidence and strong scientific rationale, we prioritised a subset of eight genes for further investigation. The selection criteria included: (a) genes previously implicated in treatment response modulation in other cancer types; (b) genes for which commercially available inhibitors exist; and (c) genes with uncharacterised functions that are known components of protein complexes involved in DNA repair. This approach allowed us to identify both novel, high‐risk candidates and more conventional targets with potential to act as sensitisers in CRC. For individual validation, we generated a polyclonal HT29 cell line with a stable single KD for each gene. The viability of these KD cells was assessed after a 24‐h FUIRI treatment across a range of concentrations, starting from low doses similar to the used in the LOF screening and increasing up to, and beyond, the previously calculated IC_50_, to rule out potential targets. The efficiency of the KD was assessed by measuring the decrease in RNA and protein levels in HT29 EcoR cells infected with each specific shRNA. Following this approach, a total of 8 genes (60% of the candidates) were positively validated (Figure S4A,B). The best results were obtained for *ATR, MORF4L2, SMARCA5* and *PARG*. Poly(ADP‐Ribose) Glycohydrolase (PARG) is the primary enzyme responsible for degrading poly(ADP‐ribose) (PAR). Following DNA damage, PARG removes PAR chains from proteins involved in DNA repair, facilitating this process. Moreover, since PAR degradation generates AMP, PARG plays a role in cellular energy balance and other key processes, including cell metabolism, apoptosis, genomic stability and transcription regulation. Whilst pharmacological inhibitors are already available for both ATR^18^, ^19^, ^20^ and PARG,^21^, ^22^, ^23^, ^24^ due to the limited scientific evidence on the potential synergism between PARG inhibitors and the FUIRI combination—unlike ATR inhibitors—we decided to continue our study with PARG.

In summary, our genetic LOF screen of chromatin regulators identified several genes affecting sensitivity to FUIRI in HT29 cells, specifically highlighting PARG as a potential drug target for combinatorial treatments.

### 3.2 *PARG* editing enhances DNA damage, sensitivity, and apoptosis induction following FUIRI and, more pronouncedly, 5FU treatment

**FIGURE 2:**
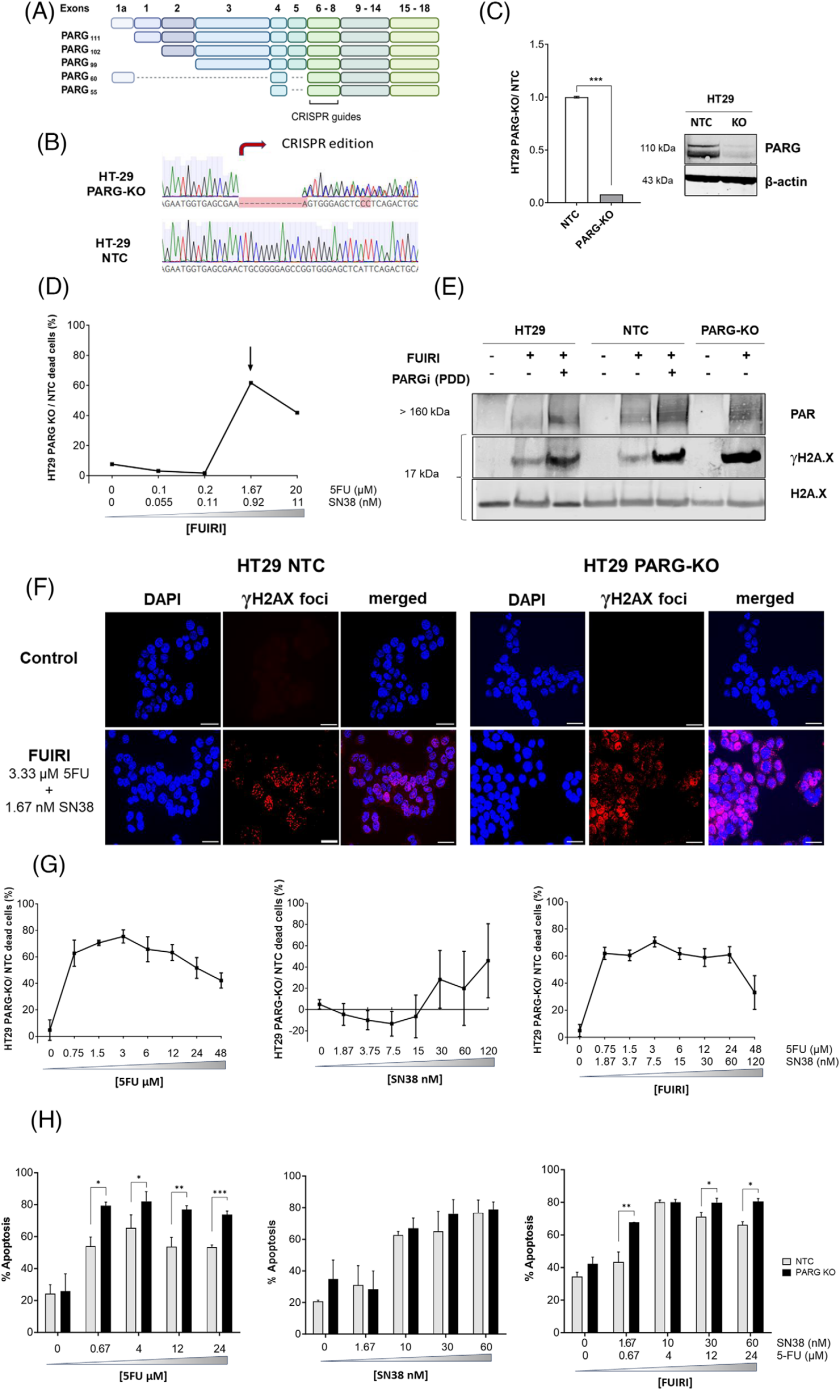
CRISPR/Cas9‐based PARG gene editing and functional validation in HT29 cells. (A) Schematic representation of human PARG isoforms. The human PARG gene undergoes alternative splicing to generate multiple isoforms with distinct domain compositions and subcellular localisations. PARG_111_ is the largest and predominantly nuclear isoform. PARG_102_ and PARG_99_, which lack part of the N‐terminal domain, are mainly cytoplasmic and perinuclear though they can translocate to the nucleus upon DNA damage. The mitochondrial isoforms PARG_55_ and PARG_60_ lack exon 5 and are catalytically inactive. CRISPR's guides were designed to target all annotated transcript variants, ensuring their disruption. (B) Representative Sanger sequencing results of DNA from CRISPR/Cas9‐edited HT29 PARG‐KO clones and NTC cells. (C) PARG protein expression levels relative to β‐actin in HT29 PARG‐KO and NTC (values from three determinations are represented as a mean ± SD). The insert shows a representative image of a WB. (D) Sensitivity of HT29 PARG‐KO to FUIRI. Cells were treated with the indicated concentrations of 5FU + SN38 and stained with DiOC (viable cells) and DAPI (non‐viable cells). The ratio of DAPI stained cells (non‐viable cells) between HT29 PARG‐KO and NTC was calculated for each treatment dilution. The arrow indicates the IC_50_ of the FUIRI treatment. The results of at least three independent experiments are expressed as percentages. (E) DePARylation activity and DNA damage levels in HT29 PARG‐KO cells. Parental HT29, NTC, and PARG‐KO cells were treated or not with 3.33 µM 5FU + 1.67 nM SN38 with or without 5 µM of PDD as indicated. PAR chain accumulation and DNA damage were detected by WB using antiPAR and H2A.X phosphorylated at Ser139 (γH2A.X) antibodies, respectively. The non‐phosphorylated form of H2A.X protein was used as a normaliser. (F) FUIRI‐induced DNA damage in HT29 PARG‐KO cells. Immunofluorescence staining of γH2A.X foci (red) shows increased DNA damage after 24 h of FUIRI treatment in HT29 PARG‐KO cells as compared to NTC cells. Nuclei were stained in blue (DAPI). Objective lens: 63× oil immersion. Scale bar: 30 µm. (G) Sensitivity of HT29 PARG‐KO cells to 5FU, SN38 and FUIRI. HT29 NTC and PARG‐KO cells were treated for 24 h with serial dilutions of 5FU, SN38, and FUIRI as indicated. Cells were stained with DiOC and DAPI, and cell viability (percentage of viable DiOC stained cells) was analysed by flow cytometry. Graphs represent the mean percentage of dead HT29 PARG‐KO cells relative to dead HT29 NTC cells (mean ± SD) of at least three independent experiments. (H) Treatment‐induced apoptosis in HT29 PARG‐KO cells. HT29 NTC and PARG‐KO cells were treated with different concentrations of 5FU, SN38 and FUIRI for 72 h, as indicated. Apoptosis was analysed by flow cytometry using the FITC Annexin V and propidium iodide staining. Bars represent the mean percentage of at least three independent determinations of apoptotic cells **± **SD. Differences in protein expression and cell viability were analysed using the Student's *t*‐test (*p < 0.05; **p < 0.01; ***p < 0.001). SD, standard deviation; KO, knockout; WB, western blot; NTC, non‐target control.

To perform further functional studies, we established an alternative PARG‐KO cell model utilising CRISPR/Cas9 technology to abolish *PARG* expression. Whilst both shRNA and CRISPR/Cas9 systems are valuable for functional investigations, the enhanced stability and reduced likelihood of off‐target effects of the latter influenced our decision to use this model for subsequent experiments. The CRISPR/Cas9 guide RNAs were designed to target exons 6–8 of the PARG gene (Figure 2A), which are shared by all annotated transcript variants, thereby ensuring disruption of all isoforms.^25^ The *PARG* gene was successfully edited in the exon 7 region (Figure 2B), resulting in undetectable protein staining by WB (Figure 2C). Using the same concentrations of the FUIRI combination, we observed a comparable or even greater reduction in post‐treatment cell viability (Figure 2D) compared to the KD model. However, the abolition of *PARG* expression had no impact on cell proliferation in the absence of the treatment (Figure S5).

To validate the functionality of our KO model, we investigated its dePARylation capacity following FUIRI treatment. A notable accumulation of PAR chains in HT29 PARG‐KO cells compared to parental and HT29 NTC cells after 24 h of treatment is illustrated in Figure 2E. When a PARG inhibitor (PDD) was added to the FUIRI treatment to parental and HT29 NTC cells an increase in PAR chain accumulation was observed, indicating a deficiency in DNA dePARylation capacity. Given that defects in dePARylation are associated with increased DNA damage, we further analysed the effect on H2A.X phosphorylation after FUIRI treatment using WB and immunofluorescence. Figure 2E,F shows that, after 24 h of FUIRI treatment, levels of  γH2A.X were higher in HT29 PARG‐KO cells as compared to HT29 or HT29 NTC cells, indicating an increase in cumulative DNA damage. This effect progressively increased over time (Figure S6). These results suggest an impaired DNA repair mechanism in our PARG‐KO model. We then examined the impact of *PARG* deficiency on response to FUIRI as well as to each drug individually. Our findings revealed that treatment with FUIRI led to a significant 33%–70% increase in cell sensitivity to treatment in HT29 PARG‐KO cells compared to the HT29 NTC line (Figure 2G). Notably, even at the lowest concentration of the drug combination, we observed a substantial 61% increased sensitivity with no further enhancement in effect at higher doses; efficacy remained consistent across the dosage spectrum. Treatment with 5FU exhibited an even more pronounced effect, whereas SN38 alone demonstrated a negligible impact on cell viability in the PARG‐KO model as compared to NTC cells. These results align with those observed in cell death activation measurements (Figure 2H). Treatment with 5FU led to a greater increase in the percentage of dead cells in PARG‐KO cells across all tested doses. In contrast, no differences were observed with SN38 treatment at any dose. Therefore, the effect seen after combination treatment in PARG‐KO cells is likely attributable to 5FU. To further characterise the type of cell death induced by 5FU and FUIRI, we evaluated caspase‐3 cleavage by WB in NTC and PARG‐KO cells. A 15 kDa band corresponding to cleaved caspase‐3 was detected in both cell lines after treatment with either drug. Notably, PARG‐KO cells showed a higher level of caspase‐3 cleavage upon 5FU exposure compared with NTC cells, whereas no clear difference was observed following FUIRI treatment. These results indicate that apoptosis contributes, at least partially, to the increased cytotoxicity observed in PARG‐deficient cells after 5FU treatment (Figure S7).

Given the well‐established role of SN38 as a topoisomerase I inhibitor that induces DPCs during replication, we were initially surprised by the lack of a clear effect of SN38. To further investigate this unexpected result, we examined whether PARG depletion could influence the formation or resolution of DPCs upon SN38 treatment. NTC and PARG‐KO cells were treated with SN38 at concentrations of 10 nM (1/3 IC_50_) and 30 nM (IC_50_) for 72 h. As shown in the figure, the percentage of DPCs increased in a dose‐dependent manner in NTC cells, whereas this increase was not observed in cells lacking PARG expression. At 30 nM, the difference in DPC accumulation between PARG‐KO and NTC cells was nearly significant. Although these results are not yet conclusive, they suggest that the PARG activity contributes to the accumulation or resolution of DPCs upon the SN38 treatment (Figure S8).

These findings suggest that the genetic ablation of *PARG* in HT29 cells, compromises the repair of damage induced by 5FU‐based treatments, leading to heightened sensitivity and increased apoptosis. Whilst the combination of 5FU and irinotecan also induced this effect, our data indicate that it is primarily driven by the impact of 5FU alone.

### 3.3 The combination of PDD00017273 with 5FU, SN38, or both exhibits synergistic effects in various colorectal cancer cell lines

**FIGURE 3:**
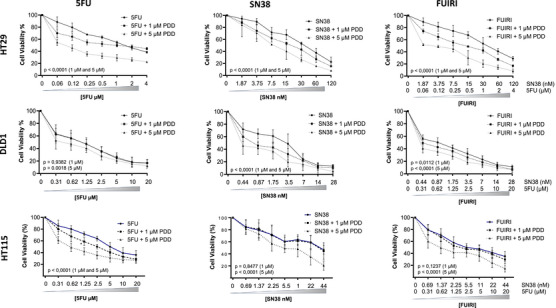
Effect of the combination of PDD with 5FU, SN38 and FUIRI on CRC cell lines. HT29, DLD1 and HT115 cells were treated with different concentrations of 5FU, SN38 and FUIRI as indicated, with or without the addition of 1 µM or 5 µM of PDD for 72 h. Cell viability was assessed using the MTT assay. Differences between treatments were analysed using a two‐way ANOVA test. The *p*‐values shown correspond to the comparison between combination treatment with PDD and chemotherapy alone. Graphs represent the mean percentage of cell viability of at least three independent experiments ± SD. SD, standard deviation. MTT, 3‐(4,5‐dimethylthiazol‐2‐yl)‐2,5‐diphenyltetrazolium bromide.

Based on our results, we aimed to explore potential synergistic interactions between a pharmacological PARG inhibitor and the administration of 5FU and SN38, either separately or in combination. Amongst the available inhibitors (see Table 1), we selected the quinazolinedione‐type PARG inhibitor PDD00017273 (PDD) for its high selectivity and efficiency in PARG inhibition, and because it had been extensively investigated in different solid tumour cell models.^26^, ^27^, ^28^ In the HT29 cell line, PDD exhibited no discernible impact on cell viability when administered as a standalone agent (Figure S9). Therefore, for subsequent synergism evaluations, we selected 1 and 5 µM doses based on their demonstrated PARG inhibitory effects in prior investigations.^26^, ^27^, ^28^ As shown in Figure 3, treatment with the lowest dose of PDD sensitised HT29 cells to 5FU, SN38 and their combination. Notably, this sensitisation effect was substantially potentiated and displayed pronounced synergy when PDD was administered at 5 µM.

Further investigations extended to the HT115 and DLD1 cell lines revealed both similarities and distinctions. In HT115 cells, PDD conferred sensitivity to SN38 and FUIRI treatment only when it was added at 5 µM, whereas sensitivity to 5FU was enhanced at both concentrations tested. However, in the DLD1 cell line, although the effect of PDD on SN38 and FUIRI is similar to that observed in the other cell lines, only the highest concentration of PDD enhanced the efficacy of the 5FU treatment. Synergism studies using the Chou and Talalay method revealed that the combination of PDD at the 5 µM concentration was synergistic in all cases and cell lines. However, when used at 1 µM, it was additive or slightly antagonistic in a few cases (Figure S10).

Based on these results, we utilised the HT29‐5FUR cell line (previously established in our laboratory^3^) to assess whether PARG inhibition could help overcome 5FU resistance. When various concentrations of 5FU were combined with PDD, we observed a significant reduction in the 5FU IC_50_ compared to treatment with 5FU alone (26 µM), which decreased to 12 µM when combined with 5 µM of PDD (Figure S11A–C). Synergy analysis using the Chou–Talalay method confirmed a stronger synergistic effect with 5 µM PDD. Notably, a synergistic response was also detected at lower 5FU concentrations when combined with 1 µM PDD (Figure S11D).

To discard any possible off‐target effect of PDD, we used both HT29 NTC and HT29 PARG‐KO models. As shown in Figure S12A–D, PDD alone did not affect cell viability, apoptosis, DNA damage, or PAR accumulation. However, when PDD was combined with 5FU in the PARG‐KO model, an increase in PAR chain accumulation was observed (Figure S12E). This accumulation was further confirmed by immunofluorescence (Figure S12F) This, however, did not translate into enhanced apoptosis, as the increase in cell death was comparable between PDD‐treated NTC cells and PDD‐treated KO cells (Figure S12G). In addition, no significant effect of the PDD treatment was observed on the DNA damage induced by 5FU or FUIRI in PARG‐KO cells. The extent of damage in HT29 NTC cells treated with PDD was comparable to that observed in HT29 PARG‐KO cells exposed to the same treatment, suggesting that pharmacological inhibition of PARG phenocopies the genetic KO in this context (Figure S12H). The observed increase in PAR accumulation may reflect incomplete KO efficiency, with residual PARG protein being further inhibited by PDD, leading to transient PAR accumulation. Altogether, these data support that the synergistic effect of PDD with chemotherapy is not due to an off‐target mechanism, but rather to specific inhibition of the PARG activity.

These results indicate that pharmacological inhibition of PARG acts synergistically with 5FU, SN38, and their combination, though the strength of this effect varies depending on the context, and suggest the potential of PARG inhibition to reverse 5FU resistance.

### 3.4 PARG‐KO tumours exhibit increased sensitivity to 5FU treatment in vivo

**FIGURE 4:**
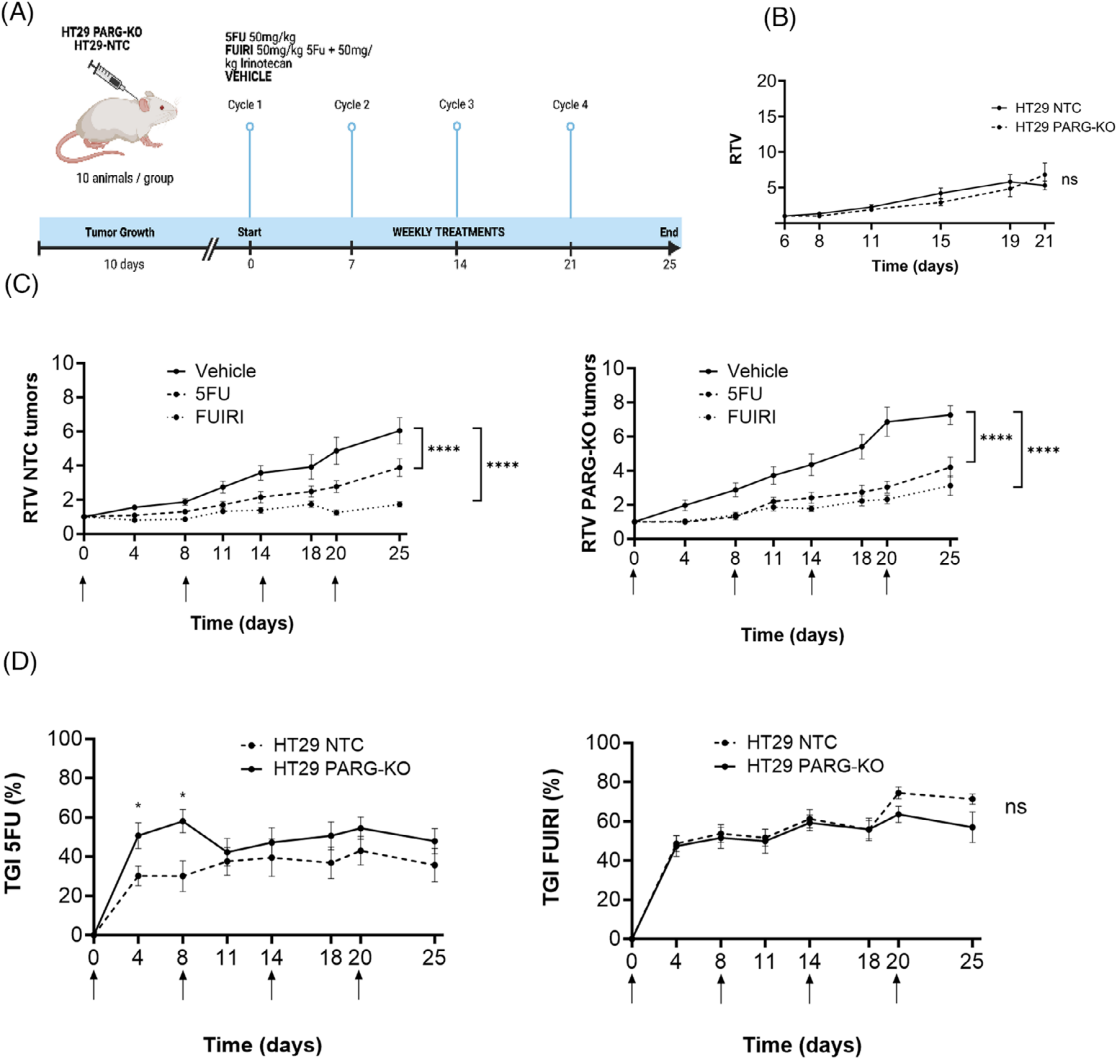
In vivo experiments. (A) Study design. HT29 NTC and PARG‐KO cells were subcutaneously injected in Balb/c mice and allowed to grow for 10 days before treatment was initiated. Tumour‐bearing mice were treated with vehicle (PBS), 5FU or FUIRI at the indicated doses (10 mice per group). Treatments were administered intraperitoneally once per week. Mice were euthanised on day 25. (B) Tumour growth. The volume of HT29 NTC and PARG‐KO subcutaneous tumours was monitored twice per week for 21 days. Measurements prior to day 6 were not possible due to the low tumour volume. The graphs represent the mean relative tumour volume (RTV: ratio between the tumour volume on day *x* and the tumour volume on day 0) ± SEM. (C) Effect of 5FU or FUIRI treatment on mice bearing HT29 PARG‐KO or NTC tumours. Tumour size was measured twice per week until the end of the experiment. Graphs show mean RTV ± SEM for the indicated treatments. (D) Tumour growth inhibition rate (TGI). The percentage of TGI induced by 5FU or FUIRI treatments in mice bearing HT29 PARG‐KO and NTC tumours, was calculated using the formula (1 − RTV) * 100. Arrows indicate the days of treatment administration for 5FU and FUIRI. Statistical significance in panels B–D were determined using a linear mixed effects (LME) model followed by a post hoc estimated marginal means (EMM) analysis (*p < 0.05). KO, knockout; SEM, standard error of the mean.

The low bioavailability of PDD makes it unsuitable for in vivo applications. However, at the time of this study, another inhibitor, COH34, showed promise, particularly in *PARP*‐mutated ovarian cancer models. We treated HT29 cells with various concentrations of COH34, both alone and in combination with 5FU, SN38, or FUIRI. Our results (Figure S13) indicated that COH34 was ineffective in all conditions. Therefore, to investigate the potential synergism between PARG inhibition and 5FU or FUIRI treatment in vivo, we subcutaneously injected HT29 PARG‐KO or NTC cells into immunodeficient mice and treated them with these drugs (Figure 4A). Tumour growth was monitored for 21 days in the absence of any treatment or drug vehicle, revealing no significant differences in RTV between both groups (Figure 4B). This observation aligns with our in vitro results, which showed no effect of PARG‐KO on cell proliferation. For the subsequent experiments, the doses of 5FU (50 mg/kg) and irinotecan (50 mg/kg), as well as the administration schedule (once a week for 4 weeks), were selected based on previous studies, the experience of our close collaborators and proving to be non‐toxic to animals (Figure S14). Notably, these doses are lower than those typically administered to patients.^29^ Both treatments reduced the growth rate (RTV) of HT29 PARG‐KO and NTC tumours with a more pronounced effect observed in the HT29 PARG‐KO tumours (Figure 4C). The tumour growth inhibition rate (TGI) indicated that 5FU had a slightly stronger effect in the absence of *PARG* expression, whilst there was no significant difference in the effect of the FUIRI treatment (Figure 4D). While these results are not definitive, they are consistent with our in vitro findings and suggest that at least, the combination of 5FU and a PARG inhibitor may be beneficial for CRC patients.

### 3.5 Investigating PARG expression as a predictive biomarker for 5FU + Irinotecan treatment in metastatic colorectal cancer patients

**FIGURE 5:**
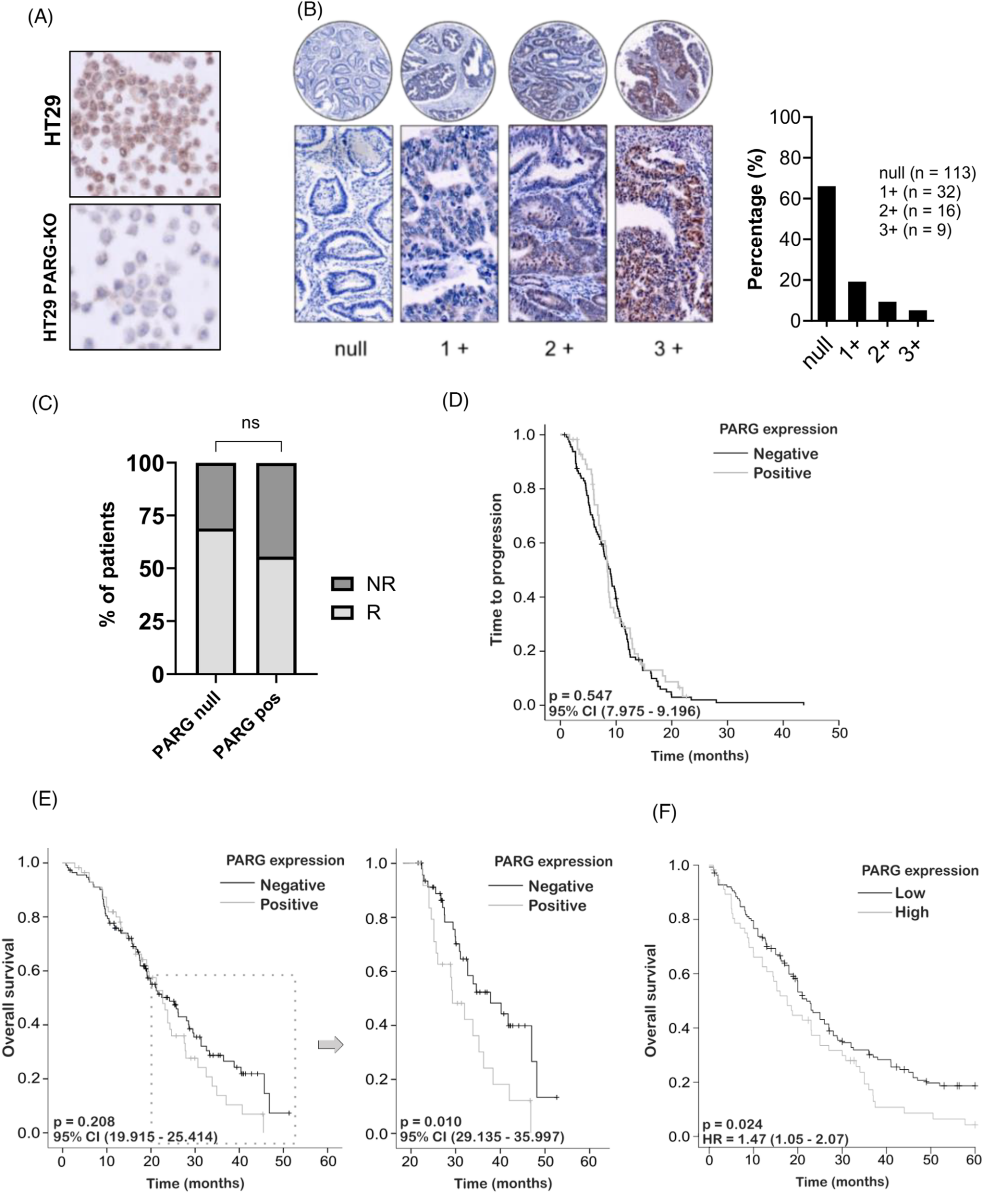
PARG expression and its association with clinical outcomes of CRC patients treated with 5FU + irinotecan. (A) PARG IHC staining. After embedding in paraffin, HT29 PARG‐KO and NTC were used as negative and positive controls for PARG IHC staining, respectively. (B) PARG IHC staining of FFPE CRC primary tumours. Representative images of CRC tissue sections showing different degrees of PARG IHC staining (left). Percentage and total number of samples in each staining category (right). (C) Response to treatment according to PARG IHC staining. Patients were categorised based on PARG IHC staining, as null or positive (1+, 2+ or 3+). The graph shows the percentage of patients responding (partial or complete response) or non‐responding (stable or progressive disease) in each of these two categories. Differences between categories were analysed using Fisher's exact test. (D) Time to progression (TTP) of CRC patients according to PARG IHC staining. The Kaplan–Meier plot shows the TTP probability for patients with null or positive PARG IHC staining. (E) Overall survival (OS) of CRC patients according to PARG IHC staining. Kaplan–Meier plot shows OS probability for patients with null or positive PARG IHC staining (left). Kaplan–Meier plot representing OS beyond 20 months according to PARG IHC staining (right). (F) OS of stage IV CRC patients according to PARG mRNA expression levels as reported by the KM plotter platform. Patients were classified as having low or high PARG mRNA expression using the best cutoff method. In panels D–F, differences between groups were calculated using the log rank test. CI, Confidence interval; HR, hazard ratio; IHC, immunohistochemistry; CRC, colorectal cancer.

Finally, we aimed to investigate whether tumour expression of PARG could serve as a predictive biomarker for treatment with 5FU + irinotecan. We utilised a retrospective cohort of 170 primary FFPE tumour samples from metastatic CRC patients treated with first‐line irinotecan + 5FU and assessed PARG protein expression via IHC. Patients’ characteristics can be seen in Table 2. HT29 PARG‐KO and NTC cells were used as negative and positive staining controls, respectively (Figure 5A) and helped us establish the positivity threshold in CRC primary tumour samples. PARG staining of tumours was classified as null, slight (1+), medium (2+) or strong (3+). We found a high percentage of samples (61%) being negative for PARG expression (Figure 5B). Therefore, to more effectively categorise the samples into homogeneous groups, we contrasted the negative cases with those exhibiting either positivity. Nearly 70% of the PARG‐negative patients responded to the treatment, whereas this percentage decreased to 56% in the positive cases, showing a trend toward statistical significance (Figure 5C) (*X* ^2^ *p* = 0.096; Fisher's test *p* = 0.115; Table 2). PARG has been associated with increased metastatic potential^30^ and indeed, we observed that patients with null PARG expression had a significantly lower number of liver metastases (≤3 metastases) compared with those expressing PARG (Figure S15). There was no difference in TTP (Figure 5D) or OS up to 20 months (Figure 5E; left) according to negative or positive PARG expression. However, a significantly higher percentage of PARG‐negative patients survived beyond 20 months compared to PARG‐positive patients (Figure 5E; right). Lastly, using the online application Kapplan Meier Plotter^31^ we observed that low *PARG* gene expression (data from GEO datasets) in stage IV CRC tumours was associated with improved OS in patients (Figure 5F). Unfortunately, data on the first‐line treatments received by these patients are unavailable. These findings suggest that the absence of PARG expression in primary tumours of mCRC patients could be indicative of a better long‐term clinical outcome when treated with combination regimens of 5FU and irinotecan. However, further research is needed to confirm its value as predictive biomarker.

**TABLE 2:** Patients’ characteristics.

Characteristic	PARG null	PARG other	*p*‐value	All
Sex, *N* (%)				
Woman	28 (62)	17 (38)	0.58	45 (26.5)
Man	65 (67)	32 (33)		97 (57)
N/A				28 (16.5)
Age, mean [range]	61.65	61.59	0.79	61.63
Localisation *N* (%)				
Colon	83 (65.4)	44 (34.6)	0.35	127 (75)
Rectum	28 (70)	12 (30)		40 (24)
Both	2 (100)	0 (0)		2 (1)
ECOG *N* (%)				
0	60 (66.7)	30 (33.3)	0.87	90 (53)
1	46 (66.7)	23 (33.3)		69 (40.5)
2	4 (57.1)	3 (42.9)		7 (4)
N/A				9 (2.5)
Metastases *N* (%)				
Liver only	54 (66.7)	27 (33.3)	0.71	81 (49)
Lung only	16 (72.7)	6 (27.3)		22 (13)
Other	4 (80)	1 (20)		5 (3)
Multiple	37 (61.7)	23 (38.4)		60 (22)
1st line *N* (%)				
FUIRI	58 (62.5)	31 (34.8)	0.75	89 (52)
FOLFIRI	55 (67.9)	26 (32.1)		81 (48)
Response *N* (%)				
R	71 (70.3)	30 (29.7)	0.115	101 (59.4)
NR	32 (57.1)	24 (42.9)		56 (33)
N/A				13 (7.6)
TTP, median [95% CI]	8.95 [7.75–10.15]	8.42 [8.02–8.82]	0.55	8.59 [7.98–9.29]
OS, median [95% CI]	24.18 [18.23–30.06]	22.66 [20.12–25.21]	0.21	22.66 [19.92–25.41]
OS > 20 months	36.45 [26.94–45.96]	27.90 [23.81–31.98]	0.01	N/A

*Notes*: PARG positive = PARG IHC staining was +1, +2 or +3; FUIRI = irinotecan (80 mg/m^2^) + 5FU (250 mg/m^2^ 48‐h infusion) weekly; FOLFIRI = irinotecan (180 mg/m^2^ on day 1) + 5FU (400 mg/m^2^ bolus and 600 mg/m^2^ 22‐h infusion) + leucovorin (LV) (200 mg/m^2^ on days 1–2) every 2 weeks. Median TTP and OS were estimated using the log‐rank test. *P*‐values were considered statistically significant at *p* > 0.05.

Abbreviations: R, responders (complete + partial response); NR, non‐responders (stable + progressive disease); TTP, time to progression; OS, overall survival; CI, confidence interval; N/A, not applicable.

## 4 DISCUSSION

CRC is a globally prevalent disease, with chemotherapy achieving response rates of approximately 50% in metastatic cases. However, resistance to treatment often leads to disease progression, underscoring the need for new therapeutic strategies. To address this challenge, researchers are investigating chromatin regulator genes, which modulate DNA accessibility and influence treatment response, as potential drug targets.

Through a high‐throughput LOF screen, we identified PARG as a key regulator of CRC treatment sensitivity. PARG plays a crucial role in DNA repair by degrading PAR chains. Our findings demonstrate for the first time, that inhibiting PARG, either genetically or pharmacologically, enhances sensitivity to chemotherapy, particularly to 5FU and the FUIRI combination and suggest that PARG inhibition could be a promising strategy to improve treatment efficacy in CRC patients.

Our approach leveraged the hEPI9 shRNA library, which targets over 900 chromatin regulator genes and has been successfully used in previous LOF screenings by our group.^4^, ^32^ Amongst the top hits, we identified *ATR*, a serine/threonine protein kinase that activates checkpoint signalling in response to genotoxic stress, promoting DNA damage repair. Notably, several studies have highlighted the synergistic effects of ATR inhibitors with 5FU and irinotecan.^18^, ^19^, ^20^ Other key candidates included *ATM* and *BRCA1*, which are involved in the HR repair pathway and play crucial roles in repairing DNA double‐strand breaks.^33^, ^34^, ^35^ Additionally, we identified *PARP14* and *PARG*, whose depletion leads to DNA damage accumulation.^36^, ^37^ A GO analysis further reinforced strong associations between our candidate genes and DNA repair processes. Mechanistically, 5FU treatment inhibits TS, resulting in a disrupted nucleotide pool and the misincorporation of 5‐FdUTP or d‐UTP into DNA. This misincorporation leads to increased DNA damage, including single‐ and double‐strand breaks.^38^, ^39^ Meanwhile, SN38 inhibits topoisomerase 1, preventing the re‐ligation of DNA strands and further facilitating double‐strand break formation.^40^ Taken together, these findings validate the robustness and reliability of our screening approach, providing key insights into potential therapeutic targets for the CRC treatment.

We selected PARG as the focus of our study due to the availability of pharmacological inhibitors already in clinical trials and the limited information on its role in CRC. PARG functions as a glycohydrolase enzyme, responsible for degrading PAR chains, a process with two critical effects: (1) mono‐ADP‐ribose moieties are released and metabolised into ATP, supporting essential metabolic processes and signalling pathways; (2) longer PAR fragments (more than three ADP units) act as apoptotic signals, influencing cell fate.^25^, ^36^ As the primary regulator of protein PARylation, PARG plays a central role in the DDR, particularly in coordination with PARP1. While other hydrolases—such as TARG1, ARH1, ARH3, MacroD1 and MacroD2—contribute to this process, their impact is relatively minor.^41^

Upon DNA damage induced by chemotherapeutic agents, PARP1 recognises single‐ or double‐strand breaks, initiating PARylation and recruiting DNA damage response proteins to start the repair process. PARG subsequently dePARylates these factors, allowing their redistribution to new DNA damage sites.^25^

Therefore, PARG inhibition disrupts this repair cycle, leading to PAR chain accumulation, increased DNA damage, and impaired repair mechanisms.^42^

Indeed, we observed a significant accumulation of PAR chains and increased DNA damage in HT29 cells treated with FUIRI, whereas these effects were absent in untreated cells. Notably, these effects were even more pronounced in HT29 PARG‐KO cells or when treatment was combined with the PDD inhibitor.

Our findings align with previous studies demonstrating that PARG depletion enhances the efficacy of chemotherapeutic agents in melanoma, ovarian cancer, glioblastoma, and head and neck cancer.^43^, ^44^, ^45^, ^46^ Moreover, the literature supports a connection between increased treatment sensitivity and apoptosis, suggesting that PAR chain accumulation, due to reduced PARG activity, along with DNA damage accumulation from impaired repair mechanisms, may trigger apoptosis.^36^

Our data indicate that PARG inhibition enhances the sensitivity of CRC cell lines to 5FU and SN38, though the effects differ between genetic KO and pharmacological inhibition. Thus, in HT29 PARG‐KO cells, sensitivity to 5FU and 5FU + SN38 was increased (reduced viability and increased apoptosis) but sensitivity to SN38 alone remained largely unaffected. In contrast, pharmacological inhibition using PDD increased sensitivity not only to 5FU, but also to SN38 and 5FU + SN38 combination across HT29, HCT115, and DLD1 cell lines.

A key factor influencing this difference may be the presence of gain‐of‐function (GOF) *TP53* mutations (R273H in HT29, S241F in HT115 and DLD1), which are known to promote chemoresistance and adaptation to replication stress.^47^ In PARG‐KO HT29 cells, mutant TP53 may activate compensatory repair mechanisms, such as alternative pathways for resolving TOP1‐DPCs, as shown in Figure S8, reducing the expected sensitivity to SN38. Conversely, acute pharmacological PARG inhibition prevents such adaptation, leading to persistent PARylation accumulation and impaired TOP1‐DPC degradation, thereby enhancing SN38 cytotoxicity.^48^

These findings were further validated in vivo, where HT29 parental and PARG‐KO cells were subcutaneously implanted in athymic mice and treated with 5FU or 5FU + irinotecan. The results showed that the 5FU treatment was more effective in reducing tumour growth in PARG‐KO tumours than in parental tumours whilst the 5FU + irinotecan treatment showed no significant differences between the two groups, suggesting the activation of compensatory mechanisms under these conditions.

However, some limitations must be considered here, such as the use of subcutaneous instead of orthotopic tumours and the reliance on a KO model rather than a pharmacological inhibitor.

This is the first study to suggest a potential synergy between PARG inhibitors, 5FU, and possibly SN38, whether administered individually or in combination, in CRC. Whilst no previous studies have explored the pharmacological effect of combining PARG and topoisomerase 1 inhibitors in cancer, two studies led by Dr. Jonathan R. Brody have reported a synergistic effect between PARG inhibitors and 5FU or CF10 (a next‐generation fluoropyrimidine) in pancreatic ductal adenocarcinoma.^49^, ^50^ Nearly all CRC patients receive 5FU at some point in their treatment, whether in the adjuvant setting or for advanced disease. Every therapeutic combination includes this fluoropyrimidine or one of its derivatives, such as capecitabine. Even in patients who develop resistance to oxaliplatin‐ or irinotecan‐based regimens, 5FU remains a cornerstone of subsequent treatment lines. Importantly, here we show in vitro, that PARG inhibition can also overcome the acquired resistance to 5FU. Therefore, our findings suggest that combining 5FU with a PARG inhibitor could have a significant clinical impact, offering a novel therapeutic strategy that enhances existing treatment options in CRC.

Finally, we explored the potential of PARG as a predictive biomarker for first‐line treatment with 5FU and irinotecan‐based combinations. Our results suggest a possible association between PARG positivity (assessed by IHC) and lower response rates as well as reduced long‐term clinical benefit. However, the lack of correlation with time to progression makes these findings inconclusive.

A key limitation here is that tumour samples were obtained from primary tumours at diagnosis, rather than from the metastases of treated patients. As a result, we cannot determine whether PARG expression remained stable or changed during tumour progression. It is possible that PARG expression increases during metastasis, meaning that patients initially classified as PARG‐negative may have actually developed PARG positivity over time. This could explain the lack of significant differences between groups. While our study does not address this directly, it is noteworthy that the work of Wang JQ et al. demonstrated a functional role for PARG in metastasis: silencing PARG in CT26 mouse CRC cells impaired their ability to form metastases after intrasplenic injection in immunocompetent mice.^51^ Although this does not imply changes in expression, it supports the idea that PARG activity may be relevant in the metastatic context. Furthermore, our own results indicate that 78% of patients with PARG‐negative tumours had 3 or fewer liver metastases, whilst this percentage was lower (58%) in those with PARG expression (Fisher's test *p* = 0.01). Therefore, further studies in larger cohorts using metastatic samples are needed to validate this hypothesis.

Notably, our findings showing a long‐term benefit in patients with PARG‐negative tumours were replicated in an independent cohort. Additionally, similar associations have been reported in other cancer types, including hepatocellular carcinoma and breast cancer.^52^, ^53^

Currently, two PARG inhibitors are in clinical trials. ETX‐19477 (858 Therapeutics) entered a Phase I trial in May 2024 to evaluate safety, pharmacokinetics (PK), pharmacodynamics (PD), and antitumor activity. Preclinical studies demonstrated inhibition of proliferation and increased γH2AX levels across multiple cancer cell lines, with in vivo efficacy in breast and ovarian xenograft models.^23^, ^54^ IDE‐161 (IDEAYA) initiated a Phase I trial in April 2023, investigating PARG inhibition in combination with immunotherapy. The study assesses the safety, tolerability, PK, PD, and antitumour activity of IDE‐161 alone or with pembrolizumab (anti‐PD‐1) in patients with BRCA1/2 mutations or HR defects. Additionally, IDEAYA is planning a new Phase I trial combining IDE‐161 with pembrolizumab for patients with MSI‐high and MSS endometrial cancer.^24^, ^55^

In conclusion, our findings open a promising avenue for clinical research, potentially marking a significant advance in the treatment of metastatic CRC. The absence of new first‐line approvals for over two decades primarily due to the failure of immune checkpoint inhibitors in MSS patients and PARP1 inhibitors highlights the urgent need for novel therapeutic targets. Our results could contribute meaningfully to enhancing both survival and quality of life for patients with this challenging malignancy.

## AUTHOR CONTRIBUTIONS

**Cristina Queralt**: Conceptualization; investigation (in vitro experiments); formal analysis; writing—original draft; writing—review & editing. **Cristina Moreta‐Moraleda**: Investigation (in vitro experiments); formal analysis; writing—original draft; writing—review & editing. **Marta Costa**: Investigation (in vivo experiments); formal analysis; writing—review & editing. **Ferran Grau‐Leal**: Investigation (in vitro experiments); formal analysis; writing—review & editing. **Jeannine Diesch**: Formal analysis; writing—review & editing. **Carla Vendrell‐Ayats**: Investigation (in vitro experiments); writing—review & editing. **Eva Musulén**: Investigation (patient sample experiments); formal analysis; writing—review & editing. **Roni H. G. Wright**: Investigation (in vitro experiments); writing—review & editing. **Cristina Bugés**: Resources; writing—review & editing. **José Luis Manzano**: Resources; writing—review & editing. **Sara Cabrero‐de las Heras**: Investigation (in vitro experiments); writing—review & editing. **Johannes Zuber**: Resources; writing—review & editing. **Marcus Buschbeck**: Funding acquisition; writing—review & editing. **Sonia Forcales**: Funding acquisition; supervision; formal analysis; writing—review & editing. **Eva Martínez‐Balibrea**: Funding acquisition; supervision; conceptualization; formal analysis; writing—original draft.

## CONFLICT OF INTEREST STATEMENT

The authors declare no conflicts of interest. All authors have read the journal's authorship agreement and policy on disclosure of potential conflicts of interest.

## ETHICS STATEMENT

All experimental procedures involving human samples, in vitro work, and animal studies were conducted in accordance with institutional, national, and international ethical standards. The use of patient samples was approved by the Clinical Research Ethics Committee (CEIC) of Hospital Germans Trias i Pujol under protocols PI‐17‐085 and PI‐19‐105. Written informed consent was obtained from all participants, and all samples were anonymized prior to analysis, in compliance with the Declaration of Helsinki.

All animal experiments were approved by the Animal Ethics Committee (CEA) of the IGTP and the Generalitat de Catalunya (project number 11730), and were performed in accordance with Directive 2010/63/EU on the protection of animals used for scientific purposes and all applicable institutional guidelines.

## DECLARATION OF GENERATIVE AI AND AI‐ASSISTED TECHNOLOGIES IN THE WRITING PROCESS

During the preparation of this work, the authors used ChatGPT 3.5 to improve readability and language. After using this tool, the authors reviewed and edited the content as needed and take full responsibility for the content of the publication.

## Supporting information



Supporting information

Supporting information
